# Religious Affiliation and Spiritual Practices: An Examination of the Role of Spirituality in Alcohol Use and Alcohol Use Disorder

**DOI:** 10.35946/arcr.v38.1.07

**Published:** 2016

**Authors:** Katie Witkiewitz, Elizabeth McCallion, Megan Kirouac

**Affiliations:** Katie Witkiewitz, Ph.D., is an associate professor; Elizabeth McCallion, M.S., and Megan Kirouac, M.S., are graduate students, all in the Department of Psychology, University of New Mexico, Albuquerque, New Mexico

“And be not drunk with wine, wherein is excess; but be filled with the Spirit” (Ephesians 5:18).

Religious affiliation, spirituality, and spiritual practices often have been studied as protective factors in the prevention and treatment of hazardous alcohol consumption (defined as drinking at a level that causes significant problems in functioning or that increases potential harms) and alcohol use disorder (AUD). Specifically, researchers have been interested in whether spirituality and spiritual practices, commonly associated with personal transformation, may also help in personal transformation of substance use behaviors. Personal transformation may involve elements— such as mindfulness and acceptance of a problem—that form the bases of behavioral treatments for substance use disorders, including AUD. Therefore, researchers are interested in whether spirituality can have a positive influence on AUD recovery.

This sidebar reviews some of the recent research that evaluates the role of spirituality in the etiology, maintenance, and treatment of hazardous drinking and AUD, as well as the efficacy of spiritual practices, including meditation and prayer, in reducing alcohol use and preventing relapse following treatment for an AUD. It also discusses results from qualitative studies that have examined life experiences and spirituality as key sources of support among individuals who have recovered from an AUD. Finally, it mentions opportunities for integrating spiritual beliefs and practices into existing empirically supported treatments for hazardous drinking and AUD.

## Spirituality and the Development of AUD

The importance of religiosity, religious experiences, and spiritual practices in the etiology and maintenance of AUD has been cited in the research literature for over 70 years (with seminal publications by Rice 1942 and Seliger 1947). Early publications described the potential benefits of religious practices in the treatment of “alcohol addiction” (Rice 1942, p. 393), although others noted that religious affiliation and early religious activity (e.g., attending church with parents) were not entirely protective against the development of AUD (Shalloo 1941; Walters 1957).

In an attempt to identify aspects of spirituality that may more or less protect against hazardous drinking and the development of AUD, more recent work has disentangled various dimensions of spirituality (e.g., Kendler et al. 2003) and identified potential mediators of the association between such dimensions and alcohol use (e.g., Drerup et al. 2011; Johnson et al. 2008). For example, Kendler and colleagues (2003) used a factor analysis of 78 items drawn from numerous sources to identify the following seven dimensions of spirituality that individuals engage in: (1) general religiosity; (2) social religiosity; (3) involvement of God; (4) forgiveness; (5) God as judge; (6) unvengefulness; and (7) thankfulness. Each was uniquely associated with the occurrence of a variety of psychiatric disorders in the general population. Of relevance to the current article, Kendler and colleagues (2003) found that greater general religiosity, social religiosity, belief in the involvement of God in a person’s life, belief in God as judge, and thankfulness all were significantly associated with a decreased risk for alcohol dependence.

Studies also have examined mediators of the associations between spirituality and alcohol use. For example, alcohol beliefs (including drinking motives and alcohol expectancies), social influences, and spiritual well-being all have been shown to significantly mediate the association between religious involvement and alcohol use among college students (Galen and Rogers 2004; Johnson et al. 2008) and among adults in the general population (Drerup et al. 2011). Alcohol use attitudes also mediate the association between religiosity and the frequency of alcohol use among adolescents such that higher rates of religious behavior were related to lower levels of alcohol use (Vaughan et al. 2011). In addition, degree of religiosity has been shown to moderate the association between perceived drinking norms and alcohol use, suggesting that greater focus on religion may buffer the effects of perceived drinking norms on heavy alcohol use behavior (Neighbors et al. 2013). Thus, involvement in religious activities or communities may exert a preventive influence on people, tempering the impact of other societal attitudes or pressures.

## Religiosity and Spirituality in Recovery From AUD

In addition to the aforementioned associations between reduced alcohol use and religion and spirituality, some evidence suggests that religion and spirituality may be associated with recovery from AUD for some individuals. For example, Sobell and colleagues (1993) interviewed individuals who had successfully recovered from substance use disorders and identified 10 major themes that were key to their success. One of these was having had a religious experience (Cunningham et al. 1994; Sobell et al. 1993). Cunningham and colleagues (1994) asked individuals at two different treatment facilities if each of these 10 themes preceded their own recovery and to rate how important each was to their decision to seek treatment. Although other reasons for seeking treatment (e.g., weighing the pros and cons of continued alcohol use) were more frequently endorsed by participants, those who cited a religious experience as a reason for seeking treatment rated that experience as just as important as other reasons behind their decision (i.e., approximately a 4 out of 5 in degree of importance); (Cunningham et al. 1994). Although these findings were reported two decades ago, they represent seminal work. More recent research has found similar associations between spiritual experiences and religiosity and AUD recovery (e.g., Dawson et al. 2012; Matzger et al. 2005). Thus, for some people, religious experiences may be an important aspect of treatment seeking and AUD recovery.

Underscoring the potential power of religious and spiritual experiences for some individuals, research has identified such experiences as important among individuals who recover from AUD and other substance use disorders without treatment. For example, Tuchfeld (1981) examined intensive interviews with 51 individuals who had spontaneously remitted from AUD. Among 13 of these individuals, “religious conversion or experience” was a factor associated with their resolution of AUD (p. 632). Similarly, Ludwig (1985) examined interviews among 29 individuals with AUD and found that “spiritual–mystical experiences” were associated with the initiation of abstinence from alcohol among individuals who recovered from AUD without treatment (p. 53). Finfgeld (2000) reviewed extant qualitative findings on self-resolution of substance use problems and found that a common theme of recovery was a reinvestment in oneself that often included involvement in religious activities.

More recently, Matzger and colleagues (2005) collected data from 659 adults with AUD and evaluated the reasons they gave that were associated with reduced drinking and sustained remission from problem drinking. Among both general-public and treatment-seeking participant groups, “undergoing a spiritual awakening” (p. 1637) was one of the predictors of sustained remission. Accordingly, existing research suggests that religious and spiritual experiences are associated with AUD recovery among both treatment-seeking and non–treatment-seeking populations.

## Changing Spirituality to Reduce Alcohol Use and Treat AUD

The majority of research conducted on spirituality and alcohol use has been focused on engagement in 12-step programs, such as Alcoholics Anonymous (AA). The AA program holds the belief that recovery is reached through spiritual experiences and an awakening to one’s higher power. To better understand the relationship between AA and drinking, researchers have examined spiritual growth as a change mechanism in AA. In a group of new members of AA, spiritual growth was found to mediate the effects of AA on increased abstinence and decreased drinking intensity such that spiritual growth was related to increased abstinence (Tonigan et al. 2013). In another study (Kelly et al. 2011), the effect of AA attendance on improved alcohol outcomes (including abstinence and drinking intensity) was partially mediated by increases in spirituality. Importantly, greater AA involvement (defined as engaging in AA-related activities, obtaining a sponsor, etc.) has been shown to be a stronger predictor of drinking outcomes and increased spiritual experience than AA attendance alone (Krentzman et al. 2013).

Changes in a person’s spirituality also may influence drinking behavior independent of his or her AA involvement. To examine this possibility, researchers have measured changes in spirituality and religious participation among individuals with AUD both with and without AA involvement (Robinson et al. 2011). Results indicated that, independent of AA involvement, 6-month increases in private spiritual or religious practices and forgiveness of self were the strongest predictors of improved drinking outcomes. Changes in daily spiritual experiences, purpose in life, a general measure of forgiveness, and negative religious coping (defined as conflict, question, and doubt regarding issues of God and faith) were also significantly associated with drinking outcomes. The findings suggest that spirituality operates independently of a 12-step framework. Therefore, looking broadly at spiritual practices—both within and outside of AA—could help researchers understand what life changes people are making when they increase their spiritual involvement that help them experience sustained improvements in their drinking practices.

Recent research, for example, has focused on the utility of mindfulness- and acceptance-based interventions for the treatment of AUD, as these have been identified as elements of personal spiritual transformation. In particular, a growing area of research supports the use of mindfulness meditation for treating substance use disorder (Witkiewitz et al. 2014; Zgierska et al. 2009). Mindfulness-based relapse prevention (MBRP), an after-care intervention for substance use disorders that incorporates mindfulness practices with relapse prevention methods, has been shown to decrease substance use, heavy drinking, and substance-related problems significantly as compared with treatment as usual and standard relapse prevention in three randomized clinical trials (Bowen et al. 2009, 2014; Witkiewitz et al. 2014). Further, Garland and colleagues (2010) developed a program for AUD called mindfulness-oriented recovery enhancement involving using mindfulness meditation practices to reduce relapse. The group found significant effects of intervention in changing cognitive, affective, and physiological responses that often are predictive of alcohol relapse following treatment. Additional research has found that significant improvements in elements of spiritual growth such as acceptance and attentional awareness—defined as the ability to attend to what one deems relevant—as well as changes in the management of craving and negative affect, significantly mediate effects of mindfulness based interventions on substance use and related outcomes (Elwafi et al. 2013; Witkiewitz and Bowen 2010; Witkiewitz et al. 2013). Only a few studies have examined the association between mindfulness practices and spiritual gains (Amaro et al. 2010), so this is an area in need of future research.

## Conclusion

References to the importance of spirituality in protecting individuals from excessive drunkenness date back to early religious texts and have been part of the research literature on harmful drinking and AUD since the early 1940s. Over the past 70 years, we have learned that religiosity and religious affiliation are not sufficient to protect against the development of AUD, but that spiritual experiences and spiritual practices, including prayer and mindfulness meditation, may be helpful in reducing hazardous drinking and in the treatment of AUD. Although AA affiliation and involvement has long been associated with the importance of spirituality in recovery, research on spirituality in AUD is not limited to AA. In recent years, increasing numbers of studies have used experimental designs to examine the effects of spiritual practices on alcohol use and AUD recovery, demonstrating that engaging in prayer may help reduce hazardous alcohol use (Lambert et al. 2010) and that engaging in mindfulness meditation practices reduces risk for relapse following treatment for AUD (Bowen et al. 2009, 2014; Witkiewitz et al. 2014). Future research should continue to examine methods of reducing hazardous alcohol use and improving outcomes in the treatment of AUD through spiritual practices.
